# Saikosaponin-d increases the radiosensitivity of smmc-7721 hepatocellular carcinoma cells by adjusting the g0/g1 and g2/m checkpoints of the cell cycle

**DOI:** 10.1186/1472-6882-13-263

**Published:** 2013-10-12

**Authors:** Bao-Feng Wang, Zhi-Jun Dai, Xi-Jing Wang, Ming-Hua Bai, Shuai Lin, Hong-Bing Ma, Ya-Li Wang, Ling-Qin Song, Xiu-Long Ma, Ying Zan, Wei-Li Min, Yan-An Cheng

**Affiliations:** 1 Department of Oncology, Second Affiliated Hospital of Medical School, Xi’an Jiaotong University, Xi’an 710004, P.R. China; 2 Department of Infectious Disease, Second Affiliated Hospital of Medical School, Xi’an Jiaotong University, Xi’an 710004, P.R. China

## Abstract

**Background:**

Saikosaponin-d (SSd), a monomer terpenoid purified from the Chinese herbal drug Radix bupleuri, has multiple effects, including anticancer properties. However, the effect of SSd on tumors exposed to radiation is largely unknown. To investigate the radiosensitizing effect of SSd and its possible mechanism, we combined SSd with radiation therapy to treat SMMC-7721 hepatocellular carcinoma cells under oxia and hypoxia.

**Methods:**

Cell growth, apoptosis, and cell cycle distribution were examined after treatment with SSd alone, radiation alone, and their combinations under oxia and hypoxia. The protein and mRNA levels of p53, Bcl2, and BAX were measured using western blot analysis and RT-PCR, respectively.

**Results:**

Treatment with SSd alone and radiation alone inhibited cell growth and increased apoptosis rate at the concentration used. These effects were enhanced when SSd was combined with radiation. Moreover, SSd potentiated the effects of radiation to induce G0/G1 arrest in SMMC-7721 cells, and reduced the G2/M-phase population under hypoxia. However, under oxia, SSd only potentiated the effects of radiation to induce G0/G1 arrest, but not G2/M-phase arrest. These effects of SSd alone, radiation alone, and their combination, were accompanied by upregulated expression of p53 and BAX and downregulation of Bcl2 expression under oxia and hypoxia.

**Conclusion:**

SSd potentiates the effects of radiation on SMMC-7721 cells; thus, it is a promising radiosensitizer. The radiosensitizing effect of SSd may contribute to its effect on the G0/G1 and G2/M checkpoints of the cell cycle.

## Background

Primary hepatocellular carcinoma (HCC), which comprises 90% of all malignant tumors that develop in the liver, is one of the most devastating human malignancies: it can cause death within a few months unless treated properly [1,2]. Early diagnosis and treatment through surgical resection or transcatheteral arterial chemoembolization (TACE) significantly improves the patient survival rate [3]. However, a substantial number of patients with HCC are unsuitable for surgery or TACE, and must be treated with using alternative methods. Radiotherapy has long been used in cancer therapy and it is frequently used to treat patients with HCC [4,5]. Oxic conditions are important for maximizing the response of cancer cells and tissues to radiation therapy. However, hypoxia is a common feature of the solid human tumor, which causes resistance to radiation in cancer cells during radiation therapy.

To overcome the hypoxic resistance, several approaches have been developed over several decades to alter the hypoxic status of cancer cells during radiation therapy. Numerous new hypoxic radiosensitizers have recently been developed and some have even been clinically evaluated [6]. Many herbs and other botanical formulations are also constantly being developed into radiosensitizers or hypoxic sensitizers [7]. However, the clinical utility of radiosensitizers remains disputed.

Saikosaponin-d (SSd), an extract from the traditional Chinese herb *Bupleurum chinensis* DC, reported exhibits anti-inflammatory [8,9], hepatoprotective [10,11], anti-angiogenic [12], and anti-cancer properties [13-16]. Our recent clinical practice of combining SSd administration with radiation in treating patients with hepatocellular carcinoma revealed that this joint treatment was more effective than either monotherapy alone, indicating a contributory effect of SSd on radiotherapy. However, the mechanism underlying radiosensitization effect of SSd on HCC cells remains unclear. To investigate the radiosensitizing effect and therapeutic efficacy of SSd, we combined SSd with radiation therapy to treat SMMC-7721 HCC cells under oxic and hypoxic conditions.

## Methods

### Cell culture

The cells were cultured in RPMI-1640 medium (PAA Laboratories GmbH, Austria) supplemented with 10% fetal bovine serum (FBS), 100 units/mL penicillin G, and 100 μg/mL streptomycin sulfate (GIBCO, Invitrogen) under a humidified 5% CO_2_ atmosphere at 37°C, and passaged once per 2 d to 3 d. During the logarithmic growth period, the cells were collected by 0.25% trypsin digestion. After counting the living cells, the cell density was adjusted to 5 × 10^4^ /mL, and then propagated by seeding into 96-well plates for further treatments, including oxic and hypoxic incubation. The oxic culture was performed by incubating the cells in RPMI-1640 medium with 10% FBS at 37°C. For hypoxia induction, the cells grown to 80% to 90% confluence were trypsinized and counted, and seeded into a 6-well plate (5 × 10^3^cells/well), followed by incubation with 100 μM cobalt chloride (CoCl_2_) for 4 h in 4 mL of serum-free medium before X-ray irradiation at a dose rate of 400 cGy/min (Clinac 2100EX; Varian Medical Systems Inc., CA) [17,18]. All of the experimental procedures were conducted in accordance with the Guide for the Care and Use of Laboratory Animals of the National Institutes of Health. Experimental protocols were approved by the Animal Care and Use Regulation of Xi’an Jiaotong University (Certificate No. 22–9601018).

### SSd preparation and experimental groups

SSd and CoCl_2_ were obtained from Sigma Chemical (St. Louis, MO). SSd was dissolved in dimethylsulfoxide (DMSO, Sigma, St Louis, MO, USA) and stored at -20°C. The desired final concentrations were achieved by dilution with RPMI-1640. The SMMC-7721 cells were treated with radiation, SSd, or PX-478 (PX, Millipore, USA) alone or in combination with radiation and SSd or PX-478. Radiation was performed using a 6 MV X-ray irradiation equipment (Clinac 2100EX; Varian Medical Systems Inc., CA) at a dose rate of 400 cGy/min. The distance between the cells and the radiation source were maintained at 100 cm. SSd was administered at different concentrations. For the joint treatment, the radiation dose was set to 2 Gy, the regular clinical radiation dose that induced moderate cell apoptosis rate. SSd was added to the cultures 2 h prior to radiation. The control cultures received the carrier solvent consisting of 0.1% DMSO.

### Cell viability assay

Cell viability was measured using an MTT assay under oxic condition or CoCl_2_-induced hypoxic condition after 4 h of chemical hypoxic culture. The SMMC-7721 cells were seeded into a 96-well plate (5 × 10^3^ cells/well), and then incubated at 37°C in 5% CO_2_ for different periods of time as designed. Then, MTT solution (5 mg/mL) (Sigma, St Louis, MO, USA) was added (20 μL/well) and the cells were incubated for another 4 h. The supernatants were removed and the formazan crystals were dissolved in 200 μL of DMSO. Finally, the optical density was determined at 492 nm using a multimicroplate test system (POLARstar OPTIMA, BMG Labtechnologies, Germany). The growth inhibition rate was calculated and plotted using the average data in quadruplicate.

### FCM apoptosis assay

After treatment, the cells were harvested and the pellet was suspended in 100 μL of binding buffer according to the manufacturer’s instructions of an Annexin V-FITC/PI kit (BD Biosciences, USA), stained with 5 μL of Annexin V-FITC and 10 μL of PI, and then incubated for 15 min at room temperature. Afterwards, another 400 μL of binding buffer was added. The cells were transferred into a BD Falcon® tube, and the percentage of the apoptotic cells in each sample was analyzed via FACSCalibur MT flow cytometer (Becton Dickinson Technologies, USA). The results represent the mean ± SD of three independent experiments.

### FCM cell cycle analysis

After treatment, the cells were harvested, fixed with ice-cold 70% ethanol, and stored at -20°C until analysis. The cells were then washed in phosphate-buffered saline, and resuspended in 50 μg/mL PI solution containing 150 μg/mL RNase A for 15 min at 37°C, and analyzed through FCM using FACSCalibur MT flow cytometer (Becton Dickinson Technologies, USA) and ModFit program (Verity Software House, Topsham, MN). The data represent three independent experiments.

### Western blotting analysis

After treatment, cell lysates were prepared with RIPA buffer containing a protease inhibitor cocktail. The homogenates were centrifuged for 10 min at 20,000 × g, and the supernatants were collected. The protein concentrations in the supernatants were determined using a NanoDrop® ND-1000 spectrophotometer (Thermo Fisher Scientific Inc.). The total cell lysates were subjected to SDS–PAGE on 10% SDS–acrylamide gel. The separated proteins were transferred onto PVDF membranes (Millipore, USA) and incubated with primary antibodies against p53, bcl2, and Bax, followed by incubation with HRP-conjugated secondary antibodies (Santa Cruz, CA). Afterward, the membrane was stripped and incubated with primary anti- β-actin antibodies (Santa Cruz, USA). The bands of protein and β-actin were visualized using Thermo Scientific Pierce® chemiluminescence substrate and the optical density of the bands were detected using a CCD camera, recorded and quantified with Syngene G Box (Syngene, UK), and compared with the relative control β-actin. The analysis was conducted at least thrice for each experiment.

### Real time PCR detection

After treatment, total RNA was isolated from the SMMC-7721 cells using Trizol Reagent® (Invitrogen Life Technologies, USA) according to the instructions of the manufacturer. The quality and quantity of the RNA samples were detected using a NanoDrop® ND-1000 spectrophotometer. The cDNA was synthesized using TaqMan® Reverse Transcription Reagents (ABI Life Technologies, USA) from 1 μg of RNA. RT-PCR detection was carried out on an ABI 7300 system using an SYBR® Green PCR Master Mix kit and predesigned primer/probe pairs for P53, bcl2, Bax, and β-actin (Santa Cruz Biotechnology, Inc.). The sequences of the primers were as follows: p53 (Forward: 5′-CCACCATCCACTACAACTACAT-3′,Reverse:5′-AGGACAGGCACAAACA-CG-3′), bcl2 (Forward: 5′-CAAATGCTGGACTGAAAAATTGTA-3′, Reverse: 5′-TATT TTCTAAGGACGGCATGATCT-3′), BAX(Forward:5′-GACACCTGAGCTGACCTTG G-3′, Reverse: 5′-GAGGAAGTCCAGTGTCCAGC-3′), and β-actin (Forward: 5′-TGGCACCCAGCACAATGAA-3′, Reverse: 5′-CTAAGTCATAGTCCGCCTAGAA GCA-3′). The reaction mixture (25 μL) contains 12.5 μL of SYBR Green PCR Master Mix, 1 μL (10 μM) of each forward primer, 1 μL (10 μM) of each reverse primer, 2 μL (100 ng) of each cDNA template, and 8.5 μL of ddH2O. The thermal cycling parameters were as follows: 95°C for 10 min for Taq polymerase activation, followed by 40 cycles of 95°C for 15 s, 60°C for 1 min. Normalization and analyses of the fold changes in target gene expression were carried out with β-actin as the internal reference via the 2–ΔΔCT method [19] and the Applied Biosystems GeneAmp 5700 SDS software. The analysis was conducted at least thrice for each experiment.

### Statistical analysis

All values are expressed as the mean ± standard deviations (SD). Statistical analysis was performed with a Student’s t-test using SPSS 13.0 statistical software. Differences with P < 0.05 were considered statistically significant.

## Results

### Influence of SSd on radiation-induced growth inhibition of SMMC-7721 cells

**Figure 1:**
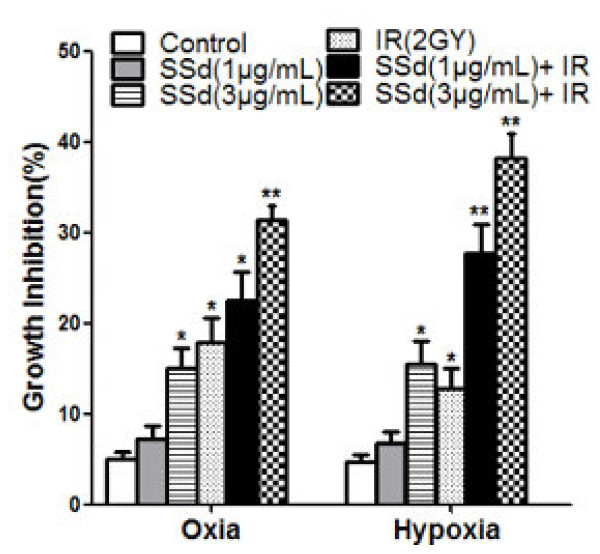
**Effects of saikosaponin-d and radiation on the growth of SMMC-7721 hepatocellular carcinoma cells.** All data are presented as mean ± SD. SSd: saikosaponin-d; IR: radiation. *P < 0.05, **P < 0.01 vs. control.

In the first experiment, cell viability was determined using an MTT assay. Under oxia or hypoxia, SSd alone and radiation alone inhibited the growth of SMMC-7721 cells. The inhibitory effect was further enhanced when SSd was combined with radiation (Figure 1 p < 0.01). We found that 1 μg/mL SSd did not significantly inhibit the hepatoma cells under oxia and hypoxia. However, combining SSd with radiation had a significantly increased inhibitory effect on HCC, and increased their sensitivity to radiation, especially under hypoxic conditions, which is more significant than that under oxic conditions. In addition, 3 μg/mL SSd was significantly more effective than 1 μg/mL SSd. These results indicate that the combination treatment induced significant cell growth inhibition in a dose dependent manner. Under oxia, irradiation followed by treatment with 1 μg/mL SSd increased the inhibition rate from 17.9 ± 3.42% to 23.1 ± 3.85%; under hypoxia, the inhibition rate increased from 12.8 ± 3.01% to 27.8 ± 4.52%. These results indicate that the combination treatment was more potent than the individual treatments, especially under hypoxic conditions.

### Influence of SSd on the radiation-induced apoptosis of SMMC-7721 cells

**Figure 2:**
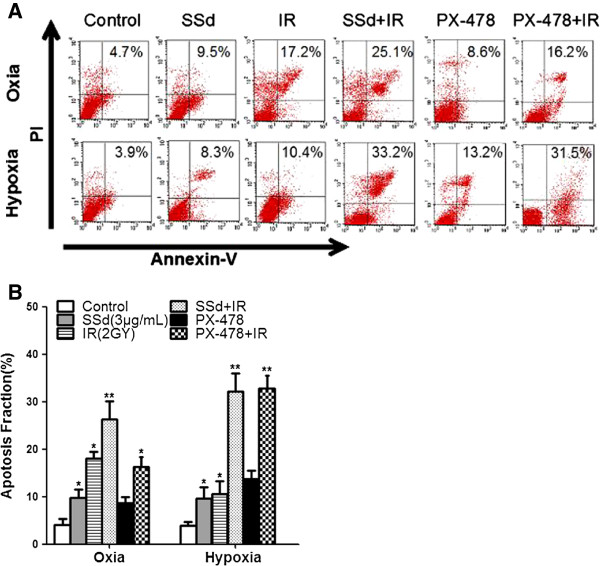
**Effects of saikosaponin-d and radiation on apoptosis in hepatocellular carcinoma cell SMMC-7721. A**. Flow cytometry shows apoptotic changes before and after treatment under oxia and hypoxia; **B**. Apoptotic fraction of cells under oxia and hypoxia; SSd: saikosaponin-d; IR: radiation. *p < 0.05, **p < 0.01 vs. control.

The apoptosis of SMMC-7721 cells induced by SSd, radiation, or their combination was determined via flow cytometry, with PX-478, an inhibitor of HIF-1α, as the positive control. The 3 μg/mL SSd treatment significantly induced the apoptosis of SMMC-7721 under both oxic and hypoxic conditions compared with the control (Figure 2A and B; p < 0.05). In addition, no significant differences were observed between oxia and hypoxia in terms of SSd-induced cell apoptosis (p > 0.05), although the cell apoptosis under oxia showed greater potential than under hypoxia. The percentage of apoptotic cells in the 2 Gy radiation treatment was higher under oxic conditions than under hypoxic conditions (Figure 2A and B, p < 0.05). Critically, the combination therapy of SSd with radiation (2 Gy) induced more apoptosis in SMMC-7721 cells under both oxic and hypoxia conditions than both SSd alone and radiation therapy alone (Figure 2A and B; p < 0.01). Hypoxic conditions enhanced the effect of radiation-induced apoptosis of the hepatoma cells more than oxic conditions in the combined treatment (Figure 2A and B; P < 0.05). Moreover, we also found that hypoxic conditions significantly enhanced the radiation-induced apoptosis of hepatoma cells was when combined with thePX-478, unlike under oxic conditions.

### SSd altered cell cycle distribution

**Figure 3:**
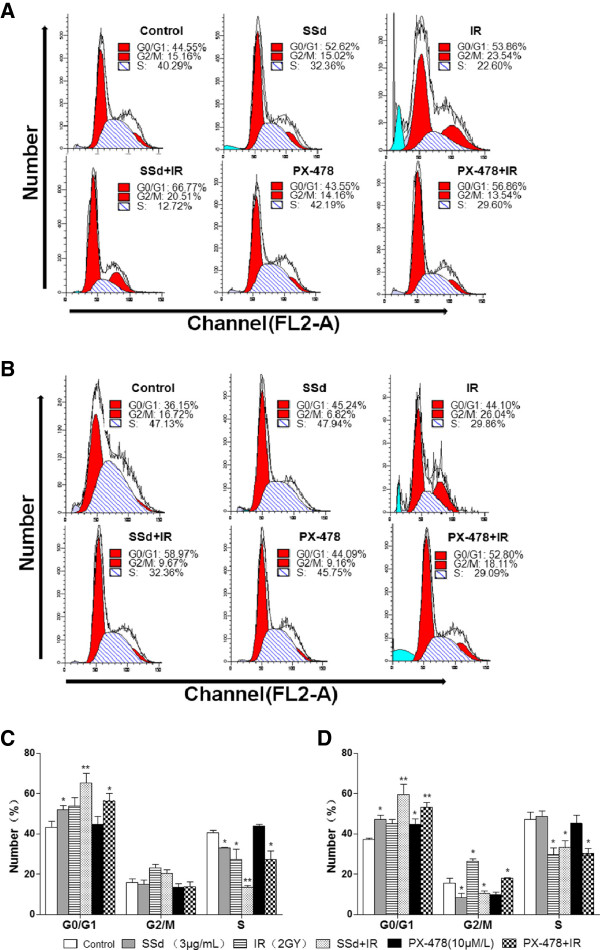
**Effect of saikosaponin-d and radiation on the cell cycle distribution of SMMC-7721 human hepatocellular carcinoma cells under oxia and hypoxia. A**. Changes in cell cycle distribution under oxia; **B**. changes in cell cycle distribution under hypoxia; **C**. Statistical analysis showing the changes in cell cycle progression before and after intervention under oxia; **D**. Statistical analysis showing the changes in cell cycle progression before and after intervention under hypoxia. SSd: saikosaponin-d; IR: radiation. *p < 0.05, ** p < 0.01 vs. control.

To determine the mechanism underlying the potentiating effect of SSd on radiation-induced apoptosis in SMMC-7721 cells, we analyzed the alteration in cell cycle using flow cytometry after every intervention. The results are shown in Figure 3. In the oxia group, the G0/G1-phase cells increased after exposure to SSd alone and radiation alone, whereas the S phase population decreased (Figures 3A and C; p < 0.05). However, the percentage of G2/M-phase cells was not significantly altered (p > 0.05). These results suggest that the G1-phase arrest decreased the S-phase population. Moreover, the SSd and radiation combination treatment increase the G0/G1 arrest and decreased the S-phase population (Figure 3A and C; p < 0.01). Under hypoxia, SSd alone, radiation alone, and their combination also induced G0/G1 arrest and decreased the S-phase population. Combined SSd and radiation treatment also further enhanced cell cycle arrest than exposure alone. Moreover, hypoxia significantly increased the effect of radiation alone on the G2/M-phase (Figures 3B and D; p < 0.05). Radiation exposure after SSd treatment almost completely abolished G2/M arrest (Figures 3B and D; p < 0.05). Meanwhile, a similar cell cycle arrest was induced under hypoxia in the PX-478 plus radiation group. In summary, the radiosensitization effect of SSd in SMMC-7721 cells under hypoxia is related to the reduction in G2/M-phase population and the increase in G0/G1 arrest, whereas the radiosensitization was related to the increase in G0/G1 arrest, but not to the decrease in G2/M phase arrest under oxia.

### Effects of SSd on the expression of radiation-induced p53 and the changes of bcl2/BAX ratio

**Figure 4:**
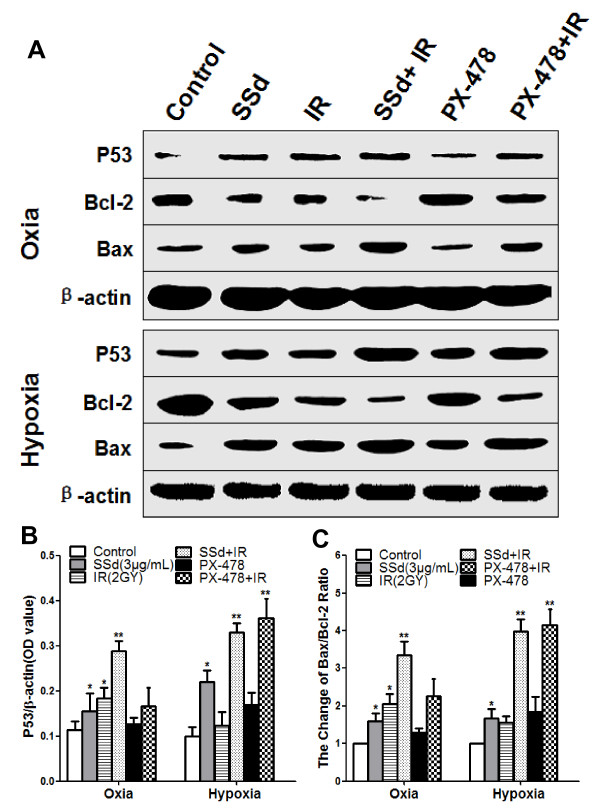
**Effect of saikosaponin-d and radiation on the p53 levels and the bcl2/BAX ratio in SMMC-7721 human hepatocellular carcinoma cells under oxia and hypoxia. A**. Western blot analysis of p53, Bax, and Bcl-2 levels under oxia and hypoxia. **B**. Relative expression of p53. **C**. Change of Bax/Bcl-2 ratio. SSd: saikosaponin-d; IR: radiation; *p < 0.05, ** p < 0.01 vs. control.

To determine whether the cell apoptosis induced by SSd, PX-478, radiation alone, and their combination is related to the p53-bcl2/BAX pathway, we investigated the p53 expression level and the bcl2/BAX ratio in the SMMC-7721 cells under oxia and hypoxia. Western blot analysis revealed that SSd alone and radiation alone significantly upregulated p53 expression and significantly decreased the bcl2/BAX ratio under oxia and hypoxia (Figure 4A–C; p < 0.05). These observations demonstrate that SSd-induced cell apoptosis may be related to the p53-bcl2/BAX pathway. However, the upregulated p53 expression and the reduction in the bcl2/BAX ratio in the SSd with radiation and the PX-478 with radiation treatments were more significant than those with SSd alone and with PX-478 alone (Figure 4A–C; p < 0.05). Therefore, SSd potentiates the response of SMMC-7721 cells to radiation at the molecular level. Moreover, PX-478 alone upregulated p53 expression and reduced the bcl2/BAX ratio under hypoxia, but not under oxia.

### Effects of SSd on radiation-induced mRNA expression of p53, BAX, and bcl2

**Figure 5:**
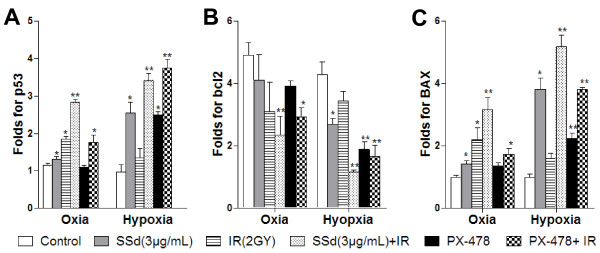
**Effect of saikosaponin-d and radiation on the mRNA levels of p53, bcl2 and BAX in SMMC-7721 human hepatocellular carcinoma cells under normoxia and hypoxia. A**. Fold changes in p53 mRNA; **B**. Folds changes in bcl-2 mRNA; **C**. Fold changes in BAX mRNA; SSd: saikosaponin-d; IR: radiation; *p < 0.05, ** p < 0.01 vs. control.

To confirm whether cell apoptosis induced by SSd, radiation, and their combination is related to the p53-bcl2/BAX pathway, we analyzed the mRNA levels of p53, bcl2, and BAX in SMMC-7721 cells under oxia and hypoxia. Under oxia, the mRNA levels of p53 and BAX were elevated after exposure to SSd alone, radiation alone, and their combinations, but not with PX-478 alone. By contrast, the bcl2 mRNA levels were decreased. Similarly, the mRNA levels of p53 and BAX increased under hypoxia, whereas bcl2 decreased with SSd alone, PX-478 alone, and their combination with radiation (Figure 5A–C; p < 0.05, p < 0.01). In addition, we also found that all treatments were more effective under hypoxia than under oxia, especially the combined treatments. Thus, these observations confirm that the cell apoptosis induced by SSd alone, PX-478 alone, radiation alone, and their combinations is related to the p53-bcl2/BAX pathway.

## Discussion

Over the past 20 years, a large number of herbs and other botanical formulations have been developed into radiosensitizers or hypoxic sensitizers, many of which have already undergone clinical evaluation and showed favorable effect with safety and lower toxicity [7]. SSd, an extract from the traditional Chinese herbs *Bupleurum chinensis* DC, has various pharmacologic properties including anti-cancer effect [13-15]. In this study, we found that incubating SMMC-7721 cells with SSd significantly decrease their cell viability under oxia and hypoxia. In addition, incubating the SMMC-7721 cells with SSd prior to irradiation further decreased cell viability at each concentration used. Treatment with 1 μg/mL SSd did not induce apoptosis in SMMC-7721 cells under oxia and hypoxia. However, treatment with SSd combined with radiation caused a more significant dose-dependent apoptosis-inducing effect, especially under hypoxic conditions. This finding implies that SSd has a potential radiosensitization effect on SMMC-7721 cells under oxia and hypoxia.

Replicating cells manifest cell-cycle progression delay in response to radiation-induced DNA damage because of activation of cell cycle checkpoints [7]. Such checkpoints at the G1–S transition and at the G2–M transition are involved in detecting and correcting DNA damage. The tumor suppressor protein p53 is a critical regulator of the cell cycle checkpoints that execute the G1/S checkpoint in response to DNA damage and induce G0/G1 arrest after irradiation [20-22]. Aside from promoting cell cycle arrest, p53 also participates in the activity of the oncogene bcl2 family to trigger apoptosis. Both Bcl2 and Bax are transcriptional targets of p53. The oncogene-derived protein Bcl2 negative controls the cellular suicide machinery pathway. The Bcl2 homologous protein Bax promotes cell death by competing with Bcl2. Although Bax–Bax homodimers act as apoptosis inducers, Bcl2–Bax heterodimer formation promotes the survival signal for cells, which induces cell cycle arrest and apoptosis in response to DNA damage [23]. Our study found that, the levels of p53 and BAX were elevated under oxia and hypoxia after exposure to SSd alone, radiation alone, and their combination, whereas the bcl2 levels were decreased. Thus, the cell apoptosis induced by SSd and radiation may involved activation of the p53/Bcl2 pathway.

To investigate the mechanism underlying the radiosensitization effect of SSd on HCC cells, we analyzed the changes in the cell cycle progression using flow cytometry after intervention. The results showed that SSd alone and radiation alone induced G0/G1-phase arrest in the SMMC-7721 cells under oxia and hypoxia, which was further enhanced by the SSd and radiation combination treatment, which is accompanied by the decrease in the S-phase population. The findings reflect the activation of the G1/S checkpoint. In addition, radiation alone induced typical G2/M phase arrest under hypoxia, which was almost completely abrogated by the combination treatment. Furthermore, we also found that the cell cycle arrest was accompanied by elevation in the mRNA and protein expression of p53 and BAX, and the decrease in Bcl2. This result may be attributed to the radiosensitization effect of SSd on hepatoma cells.

PX-478, an inhibitor of the hypoxia-inducible factor-1α (HIF1α), has potent antitumor activity against various human tumor xenografts associated with the levels of the HIF1α [24]. We found that PX-478 alone under oxia did not alter the p53 expression levels in SMMC-7721 cells. Under hypoxia, however, PX-478 alone induced apoptosis and cell cycle arrest or upregulated p53 expression. Koh et al. [25] agreed that the cell apoptosis induced by PX-478 under hypoxia may contribute to its antitumor activity against HIF-1α expression by increasing the p53 level, whereas the apoptosis induced by PX-478 does not require oxygen, pVHL, or p53.

## Conclusion

In summary, SSd increases the radiosensitivity of SMMC-7721 hepatoma cells and induces apoptosis, especially under hypoxic conditions. The radiosensitizing effect of SSd on hepatoma cells is more obvious under hypoxia, and its mechanism may contribute to its effect on the G0/G1 and G2/M checkpoints of the cell cycle. Thus, SSd may be a promising radiosensitizer. However, whether SSd alone and SSd combined with radiation generates lethal single stranded DNA breaks or reactive oxygen species requires further study.

## Competing interests

The authors declare that they have no competing interests.

## Authors’ contributions

WBF, DZJ and WXJ designed the research. LS, BMH, MXL and MHB performed the experiments throughout this research. WYL, CYA, WXJ and ZY contributed to the reagents, and participated in its design and coordination. WBF, MWL and LS analyzed the data; LS and WBF wrote the paper. All authors have read and approved the final manuscript.

## Pre-publication history

The pre-publication history for this paper can be accessed here:

http://www.biomedcentral.com/1472-6882/13/263/prepub
